# Serum Insulin-Like Growth Factor-1 Predicts Disease Progression and Survival in Patients with Hepatocellular Carcinoma Who Undergo Transarterial Chemoembolization

**DOI:** 10.1371/journal.pone.0090862

**Published:** 2014-03-04

**Authors:** EunJu Cho, Hyo-Cheol Kim, Jeong-Hoon Lee, Jeong-ju Yoo, Won-Mook Choi, Young Youn Cho, Min Jong Lee, Yuri Cho, Dong Hyeon Lee, Yun Bin Lee, Su Jong Yu, Yoon Jun Kim, Jung-Hwan Yoon, Jin Wook Chung, Chung Yong Kim, Hyo-Suk Lee

**Affiliations:** 1 Department of Internal Medicine, Kangwon National University Hospital, Chuncheon, Korea; 2 Department of Radiology, Seoul National University College of Medicine, Seoul, Korea; 3 Department of Internal Medicine and Liver Research Institute, Seoul National University College of Medicine, Seoul, Korea; Xiangya Hospital of Central South University, China

## Abstract

Insulin like-growth factor-1 (IGF-1) reflects hepatic synthetic function and plays a major role in the development and progression of various cancers. In the present study, we investigated whether baseline serum IGF-1 levels predict time-to-progression (TTP) and overall survival (OS) in hepatocellular carcinoma (HCC) patients treated with transarterial chemoembolization (TACE). A total of 155 consecutive treatment-naive patients with HCC who had undergone TACE as initial treatment were included from a prospective cohort. Baseline serum IGF-1 levels were analyzed with regard to their associations with disease progression and survival. During a median follow-up period of 41.8 months, patients with low IGF-1 levels showed significantly shorter TTP (median, 6.0 months; 95% confidence interval [CI], 4.5–7.6) than patients with high IGF-1 levels (median, 16.5 months; 95% CI, 4.9–28.1; *p* = 0.003). In the multivariate analysis, BCLC stage, serum vascular endothelial growth factorlevels, and IGF-1 levels were independent risk factors for disease progression. The hazard ratio (HR) of progression for each 10 ng/mL decrease in IGF-1 level was 1.072 (95% CI, 1.029–1.117; *p* = 0.001). Furthermore, together with tumor size, stage, and treatment response, IGF-1 levels were an independent predictor of poorer survival (for each 10 ng/mL decrease in IGF-1 level; HR, 1.057; 95% CI, 1.001–1.115; *p* = 0.045). In conclusion, low baseline IGF-1 levels independently correlated with shorter TTP and poorer OS in patients with HCC who underwent TACE.

## Introduction

Hepatocellular carcinoma (HCC) is a hypervascular tumor, and angiogenesis induced by angiogenic factors, such as vascular endothelial growth factor (VEGF), plays a pivotal role in tumor growth, invasion, and metastasis [1]. This characteristichas made transarterial chemoembolization (TACE) a main treatment for nonsurgical patients with large, multifocal tumor [2]–[4]. However, the long-term prognosis is still poor because of the high frequency of disease progression, although initial remission is achieved by TACE. Therefore, the identification of risk factors for tumor progression after TACE is important for selecting appropriate therapies and predicting a prognosis.

Insulin-like growth factor-1 (IGF-1) is a potent survival factor and implicated in the development and progression of various cancers [5]. The stimulatory ligand and its receptors are overexpressed in many cancer cells and induce cell survival, proliferation, and angiogenesis. Additionally, IGF-1 has been shown to synergize with tissue hypoxia to enhance tumor growth and metastasis [6], [7]. Moreover, epidemiologic studies have shown an association between high circulating IGF-1 levels and an increased risk of prostate, breast, and colon cancer [8], [9]. However, the relevance IGF-1 system to HCC is somewhat different from other malignancies. Because the majority of circulating IGF-1 is produced by the liver, IGF-1 levels reflect hepatic function and are inversely correlated with the severity of background chronic liver disease [10], [11]. Moreover, recent study has shown that a decrease in serum IGF-1 levels is associated with the development of HCC, regardless of the grade of hepatic dysfunction [12]. Low serum IGF-1 levels were also associated with a poor response to anti-angiogenic therapy, leading to an unfavorable prognosis in patients with advanced HCC [13]. In addition, we recently found that low baseline serum IGF-1 levels were independently associated with a shorter time to recurrence and poorer OS in patients who received curative therapy for early-stage HCC [14].

Collectively, these findings suggest that IGF-1 levels may be associated with the extent of disease, treatment response, and prognosis. However, this assumption has not been tested in patients with HCC who have undergone TACE. Thus, the present study evaluated whether basal serum IGF-1 levels can predict prognosis of HCC patients according to different risks of disease progression and death after TACE.

## Materials and Methods

### Study Population

The present study was part of an ongoing prospective cohort study identifying the biomarkers associated with treatment response and prognosis in HCC at Seoul National University Hospital (Seoul, Republic of Korea). Between January 2006 and July 2010, 198 treatment-naive patients with HCC who had undergone TACE as initial treatment were enrolled in this study. The diagnosis of HCC was made according to the recommendations of the American Association for the Study of Liver Diseases [4]. The indication of TACE was mainly non-surgical patientswith large/multifocal HCC, but patients with early-stage HCC who were unsuitable for surgical resection or ablation because of liver function, comorbidity, or technical infeasibility also received TACE. Among the enrolled patients, we excluded 43 patients because ofextrahepatic metastasis (*n* = 5), no measurable target lesions (infiltrative pattern, or largest lesion less than 1 cm) according to the modified Response Evaluation Criteria in Solid Tumors (mRECIST) [15] (*n* = 35), and early follow-up loss after first-session TACE (*n* = 3). The remaining 155 patients were included in the study.

The study protocol conformed to the ethical guidelines of the World Medical Association Declaration of Helsinki and was approved by the Institutional Review Board of Seoul National University Hospital. All of the participants provided written informed consent.

### TACE Procedure

Details of the procedure performed in our institution have been described previously [16]. Briefly, a solution that contained 10–60 mg of doxorubicin hydrochloride emulsion (Adriamycin RDF; Ildong Pharmaceutical, Seoul, Korea) and 2–12 mL of ethiodized oil (Lipiodol; Andre Guerbet, Aulnay-sous-Bois, France) was infused through a microcatheter (Microferret; Cook, Bloomington, IN, or Progreat; Terumo, Tokyo, Japan) until arterial flow stasis was achieved and/or ethiodized oil appeared in the portal branches. Subsequently, embolization was performed with absorbable gelatin sponge particles 1 mm in diameter (Gelfoam; Upjohn, Kalamazoo, MI, or Cutanplast;MasciaBrunelli, Milano, Italy) soaked in a mixture of 10–20 mg doxorubicin hydrochloride and 10 mL of nonionic contrast medium.

Contrast-enhanced dynamic computed tomography (CT) was performed 4 weeks after TACEtoassess the need of a consecutive treatment. Because mRECISTcould reliably predict the prognosis of HCC patients undergoing TACEas initial treatment [17]–[19], we assessed overall responseafter the initial TACE using mRECIST criteria. The patients with objective response (defined as complete or partial response) were classifiedas responders and the others were classifiedas non-responders. Repeated TACE was performed every 6–8 weeks if viable tumors (i.e., contrast material uptake in the arterial phase and washout in the portal and/or late venous phase) were detected on sequential imaging without deterioration of liver function.

### Measurement of Serum IGF-1 and VEGF

Peripheral blood samples were collected before the first treatment. Serum samples were aliquoted and stored at −80°C. Serum IGF-1 levels were quantified using an immunoradiometric assay with standard commercial kits (Immunotech, Marseille, France). VEGF levels were measured using a Luminex 200 (Millipore, Billerica, MA) and Bio-PlexTM Reagent Kit (Bio-Rad, Hercules, CA) according to the manufacturer’s instructions.

### Outcomes and Assessments

The primary endpoint of the study was time-to-progression (TTP), measured from the date of enrollment until the first documented tumor progression in imaging studies according to the mRECIST. The secondary endpoint was OS, defined as time from enrollment to death.

### Statistical Analyses

Continuous variables are presented as medians and interquartile ranges (IQRs) or means and standard deviations (SDs). The Mann-Whitney U test and Kruskal-Wallis test were used to analyze differences between the different groups. The *χ^2^* test or Fisher’s exact test was used for categorical data. Pearson’s and Spearman’s correlation coefficients (*r* and *ρ*, respectively) were used to evaluatethe relationships between clinical characteristics and the levels of IGF-1 and VEGF. Time-dependent receiver-operating characteristic (ROC) curves for censored survival data were constructed to define the best cut-off value for predicting outcome [20]. The best cut-off was defined as the value with the maximal sum of sensitivity and specificity [21]. Confidence intervals (CIs) for the area under the ROC curve (AUC) were obtained from 1,000 bootstrapped samples. Survival curves were compared using the log-rank test and Breslow test. Cox proportional hazard regression analysis was performed to evaluate independent risk factors for disease progression and OS. Variables with *P*<0.05 in the univariate Cox regression analysis were subjected to multivariate analysis using forward stepwise selection. Differences at *P<*0.05 were considered statistically significant. The analyses were performed using SPSS 19.0 K (SPSS, Chicago, IL) and the R statistical programming environment, Version 3.0.1 (http://www.r-project.org).

## Results

### Baseline Patient Characteristics and Treatment Outcomes

The baseline characteristics of the study population are summarized in Table 1. Of the 155 patients, 126 (81.3%) were male. The median age at the time of diagnosis was 56 years (IQR, 50–64 years). Hepatitis B virus infection was the most common cause of underlying liver disease (76.8%), and most (85.8%) of the patients had preserved Child-Pugh class A liver function. The median size of the largest measurable lesion was 3.8 cm (IQR, 2.1–7.0 cm). According to the Barcelona Clinic Liver Cancer (BCLC) staging system, 10, 63, 44, and 38 patients belonged to stage 0, A, B, and C, respectively. The median number of TACE procedures was 4 (IQR, 2–7).

**Table 1:** The levels of insulin-like growth factor-1 and vascular endothelial growth factoraccording to clinical characteristics.

	Patients (*n* = 155)^1^	IGF-1 (ng/mL)		VEGF (ng/mL)	
		Median (IQR)^2^	*P*	Median (IQR)	*P*
Age (years)			0.746		0.558
<60	90 (58.1)	94.5 (52.8–125.5)		29.13 (159.7–524.8)	
≥60	65 (41.9)	88.0 (52.5–122.5)		263.9 (150.7–505.6)	
Gender			0.813		0.579
Female	29 (18.7)	89.0 (57.5–118.0)		326.3 (150.7–600.6)	
Male	126 (81.3)	91.0 (51.3–127.8)		279.3 (158.9–465.9)	
Hepatitis infection status^||^			0.293		0.842
HBsAg positive	119 (76.8)	96.0 (54.0–130.0)		271.3 (157.1–448.4)	
Anti–HCV positive	19 (12.3)	72.0 (43.0–106.0)		285.1 (140.6–622.2)	
NBNC	17 (11.0)	91.0 (51.0–108.5)		301.7 (152.7–554.5)	
Clinical cirrhosis^3^			0.104		0.087
Present	142 (91.6)	88.0 (52.8–120.3)		268.9 (153.7–485.6)	
Absent	13 (8.4)	112.0 (68.0–145.0)		519.3 (252.6–881.9)	
Child-Pugh class			<0.001		0.436
A	133 (85.8)	99.0 (62.0–131.5)		286.0 (159.7–524.8)	
B	22 (14.2)	49.0 (20.5–71.8)		232.2 (117.5–470.6)	
Serum AFP (ng/mL)			0.829		0.232
<200	84 (54.2)	88.0 (53.2–124.0)		254.6 (157.9–420.5)	
≥200	71 (45.8)	97.0 (49.0–125.0)		324.4 (152.8–559.0)	
AJCC tumor stage^4^			0.022		0.001
T1	53 (34.2)	99.0 (65.5–150.0)		275.2 (144.2–466.1)	
T2	65 (41.9)	88.0 (54.0–132.5)		235.8 (123.8–416.4)	
T3	37 (23.9)	71.0 (40.5–106.5)		415.6 (246.6–799.6)	
BCLC stage			0.010		<0.001
0	10 (6.5)	78.0 (27.0–96.0)		247.7 (98.4–702.0)	
A	63 (40.6)	103.0 (65.8–148.8)		240.4 (142.0–335.1)	
B	44 (28.4)	74.5 (53.3–111.3)		249.8 (134.9–428.1)	
C	38 (24.5)	94.5 (41.3–109.6)		423.0 (286.8–735.0)	
CLIP			0.283		0.003
0	32 (20.6)	94.5 (65.3–150.5)		275.2 (161.1–486.0)	
1	66 (42.6)	96.5 (56.5–136.8)		235.8 (141.8–388.2)	
2	32 (20.6)	84.5 (50.3–110.5)		289.8 (123.7–505.6)	
3	18 (11.6)	78.5 (41.0–112.8)		423.0 (273.7–838.2)	
4	7 (4.5)	47.0 (32.0–109.0)		700.1 (383.4–950.2)	
Okuda stage			0.001		0.003
1	126 (81.3)	97.5 (62.0–131.3)		258.9 (151.4–435.2)	
2	29 (18.7)	48.0 (27.0–103.0)		514.5 (246.2–809.2)	

1 Data are numbers of patients. Numbers in parentheses are percentages.

2 Data are median values, with the interquartile range (IQR) in parentheses.

3 Clinical cirrhosis was defined as having either one of the following: (*i*) presence of cirrhosis-related complications including ascites, varices, encephalopathy and/or (*ii*) radiographic evidence of small-sized or nodularliver with or without splenomegaly.

4 According to American Joint Committee on Cancer staging system (7th edition, 2010).

AFP = α-fetoprotein, BCLC = Barcelona Clinic Liver Cancer, CLIP = Cancer of the Liver Italian Program, HBsAg = hepatitis B surface antigen, HCV = hepatitis C virus, NBNC = HBsAg- and anti-hepatitis C virus-negative.

During a median follow-up period of 41.8 months (IQR, 16.3–67.1 months), 132 patients (85.2%) experienced disease progression. The overall cumulative progression rate in HCC patients following TACE was 68.8% after 1 year, 83.0% after 2 years, and 87.9% after 3 years. The median time-to-progression (TTP) and median progression-free survival (PFS) were both 6.7 months (95% confidence interval [CI], 5.0–8.4). The overall cumulative death rate was 21.4% after 1 year, 32.6% after 2 years, and 42.0% after 3 years. The median OS was not reached (71 of 155 patients died).

### The Levels of Serum IGF-1 and VEGF According to Clinical Characteristics

The associations between clinical factors and the levels of IGF-1 and VEGF are described in Table 1. The levels of IGF-1 decreased in patients with Child-Pugh class B (*P<*0.001), whereas they were not significantly different according to age, gender, and etiologies of underlying liver disease. With respect to tumor factors, IGF-1 levels as a continuous levels were not significantly correlated with maximum tumor size and serum α-fetoprotein (AFP) levels, but showed a negative correlation with tumor number (*r = *−0.206, *P = *0.01). Additionally, the levels of IGF-1 showed a trend toward a decrease with advancingtumor stage, determined by the American Joint Committee on Cancer (AJCC) tumor-node-metastasis (TNM) staging (*ρ = *−0.218, *P = *0.01) and Okuda (*ρ = *−0.273, *P = *0.001). Consistent with previous reports [22], [23], serum VEGF levels showed a positive correlation with tumor size (*r = *0.430, *P*<0.001) andstage determined by various systems, such as the AJCC TNM (*ρ = *0.202, *P = *0.02), BCLC (*ρ = *0.315, *P<*0.001), the Cancer of the Liver Italian Program (CLIP) score (*ρ = *0.208, *P = *0.02) and Okuda (*ρ = *0.260, *P = *0.002).

### Serum IGF-1 Levels as an Independent Prognostic Factor for HCC Progression and Survival

**Figure 1:**
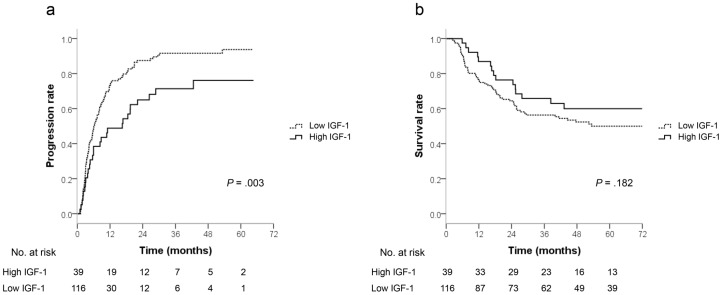
Graphs show Kaplan-Meier estimates of (a) time-to-progression and (b) overall survival in HCC patients after transarterial chemoembolization according the levels of baseline serum IGF-1. A cut-off value of 125 ng/mL was used, and*P* values werederived bylog-rank test.

IGF-1 was initially analyzed for its prognostic value as a continuous variable. Univariate Cox analyses showed thathigh Child-Pugh score, larger tumor size (≥5 cm), multiple tumors (i.e., >1), presence of portal vein thrombosis (PVT), advanced BCLC stage, increasing levels of VEGF, and decreasing levels of IGF-1 were significantly associated with shorter TTP (Table 2). Patients with high serum AFPlevels (≥200 ng/mL) showed a trend toward shorter TTP compared with patients with low AFP levels (<200 ng/mL), but the differencewas not significant (*P* = 0.08). No significant differenceswere found according to age or gender. In the multivariate analysis, BCLC stage, IGF-1 levels, and VEGF levels were independent risk factors for disease progression. The hazard ratio (HR) of progressionfor a 10-point decrease in IGF-1 level was 1.072 (95% CI, 1.029–1.117; *P* = 0.001), and the HR for a 10-point increase in VEGF level was 1.006 (95% CI, 1.002–1.010; *P* = 0.003). Time-dependent ROC analysis showed that the optimal cut-off valuefor predicting progression within 2 years was 125 ng/mL for IGF-1(sensitivity, 80.7%; specificity, 45.9%; AUC, 0.63; 95% CI, 0.52–0.75), and140 pg/mL for VEGF (sensitivity, 83.2%; specificity, 31.6%; AUC, 0.57; 95% CI, 0.46–0.68), respectively. Kaplan-Meier curves for TTP are shown in Fig. 1a. Patients with low IGF-1 levels (<125 ng/mL) showed a significantly shorter TTP (median, 6.0 months; 95% CI, 4.5–7.6) than patients with high IGF-1 levels (≥125 ng/mL; median, 16.5 months; 95% CI, 4.9–28.1; *P* = 0.003). The cumulative rates of progression at 1, 2, and 3 years were 73.3%, 87.5%, and 91.6%, respectively, in patients with low IGF-1 levels and 48.8%, 65.0%, and 71.4%, respectively, in patients with high IGF-1 levels. Together with Child-pugh score, BCLC stage, and high levels of VEGF (≥140 pg/mL), lowlevels of IGF-1 remained an independent predictor of disease progression (HR, 1.785; 95% CI, 1.050–3.034; *P* = 0.03) in multivariate analysis.

**Table 2:** Univariate and multivariate analyses of factors associated with time-to-progression.

	Univariate analysis			Multivariate analysis		
Variable	Hazard ratio	95% CI	*P* Value	Hazard ratio	95% CI	*P* Value
Age (≥60 vs. <60 years)	0.758	0.536–1.070	0.115			
Gender (male vs. female)	1.145	0.736–1.783	0.548			
Hepatitis virus infection			0.716			
NBNC	1.000					
HBV	1.218	0.696–2.132				
HCV	1.325	0.663–2.647				
Child-Pugh score	1.431	1.217–1.684	<0.001	–	–	0.155
AFP (≥200 vs. <200 ng/mL)	1.350	0.959–1.900	0.086			
Tumor size (≥5 vs. <5 cm)	2.421	1.699–3.452	<0.001	–	–	0.064
Tumor number (multifocal vs. unifocal)	1.835	1.289–2.613	0.001	–	–	0.420
Presence of PVT	3.399	2.127–5.432	<0.001	–	–	0.665
BCLC stage			<0.001			
0	1.000			1.000		
A	2.331	0.996–5.457		3.117	1.194–8.135	0.020
B	3.946	1.662–9.372		5.172	2.476–10.805	<0.001
C	13.748	5.581–33.865		22.299	6.623–75.073	<0.001
Serum IGF-1 (ng/mL)	0.995	0.992–0.999	0.007	0.993	0.989–0.997	0.001
Serum VEGF (pg/mL)	1.001	1.000–1.001	0.002	1.001	1.000–1.001	0.003

The hazard ratio was calculated by using the Cox proportional hazard model.

AFP = α-fetoprotein, BCLC = Barcelona Clinic Liver Cancer, HBV = hepatitis B virus, HCV = hepatitis C virus, NBNC = HBsAg- and ant-hepatitis C virus-negative, PVT = portal vein thrombosis.

The levels of IGF-1 were also significantly associated with OS. In the multivariate analysis, IGF-1 levels, BCLC stage, larger tumor size, and mRECIST non-responders were independent risk factors for poorer OS (Table 3). The HR of death for a 10-point decrease in IGF-1 level was 1.057 (95% CI, 1.001–1.115; *P* = 0.04). When a cut-off value of 125 ng/mL for predicting progression was used, patients with low IGF-1 levelsshowed a trend toward poorersurvival compared with patients with high IGF-1 levels, but the differencewas not significant (*P* = 0.18; Fig. 1b). However, when applyingthe best cut-off value of 56 ng/mL for predicting 5-year survival (sensitivity, 39.6%; specificity, 79.2%; AUC, 0.59; 95% CI, 0.48–0.67), patients with low IGF-1 levels had significantly poorersurvival than patients with the high IGF-1 levels, corresponding to a HR of 1.740 (95% CI, 1.140–2.656; *P* = 0.01). The median OS could not be estimated for either group because of the low number of death events. In the multivariate analysis, IGF-1 levels lower than 56 ng/mLremained a significant risk factor for poorer OS (HR, 1.889; 95% CI, 1.128–3.163; *P* = 0.02), together with BCLC stage, larger tumor size, and mRECIST non-responders.

**Table 3:** Univariate and multivariate analyses of factors associated with overall survival.

	Univariate analysis			Multivariate analysis		
Variable	Hazard ratio	95% CI	*P* Value	Hazard ratio	95% CI	*P* Value
Age (≥60 vs. <60 years)	0.875	0.582–1.316	0.522			
Gender (male vs. female)	0.824	0.503–1.350	0.443			
Hepatitis virus infection			0.601			
NBNC	1.000					
HBV	1.339	0.671–2.672				
HCV	1.070	0.435–2.633				
Child-Pugh score	1.363	1.151–1.614	<0.001	–	–	0.759
AFP (≥200 vs. <200 ng/mL)	1.985	1.316–2.994	0.001	–	–	0.331
Tumor size (≥5 vs. <5 cm)	4.750	2.927–7.709	<0.001	2.947	1.621–5.359	<0.001
Tumor number (multifocal vs. unifocal)	0.996	0.666–1.491	0.985			
Presence of PVT	3.907	2.589–5.896	<0.001	–	–	0.365
BCLC stage			<0.001			
0	1.000			1.000		
A	1.869	0.434–8.059		1.285	0.282–5.851	0.746
B	3.379	0.794–14.385		1.444	0.312–6.682	0.639
C	10.962	2.658–45.206		5.37	1.179–24.466	0.030
Serum IGF-1 (ng/mL)	0.995	0.990–0.999	0.019	0.995	0.989–1.000	0.043
Serum VEGF (ng/mL)	1.000	1.000–1.001	0.008	–	–	0.318
mRECIST non-responder	4.555	3.018–6.874	<0.001	2.607	1.522–4.465	<0.001

The hazard ratio was calculated by using the Cox proportional hazard model.

AFP = α-fetoprotein, BCLC = Barcelona Clinic Liver Cancer, HBV = hepatitis B virus, HCV = hepatitis C virus, mRECIST = modified Response Evaluation Criteria in Solid Tumors, NBNC = HBsAg- and ant-hepatitis C virus-negative, PVT = portal vein thrombosis.

## Discussion

The present study showed that decreasing levels of baseline serum IGF-1 were associated with higher progression rates as well as poorer OS in HCC patients who underwent TACE. The associations were independent of other well-known prognostic factors, including Child-Pugh score, serum AFP level, tumor size and number, stage, and baseline serum VEGF level.

The result that low serum IGF-1 levels were associated with poor prognosis appears to be paradoxical because high IGF-1 levels and subsequent activation of the IGF system have been known as relevant signaling alterations in various cancers [5]. Nonetheless, our results maintain consistency with previous studies of the levels of circulating IGF-1 and prognosis in HCC patients. Kaseb*et al*showed thatpatients with low IGF-1 levels were more likely to exhibit advanced pathologic parameters of HCC, such as multinodularity, large tumor size, and vascular invasion, and had shorter OS [24], [25]. Similarly, low baseline serum IGF-1 levels were associated with poor treatment response, PFS, and OS in patients who received anti-angiogenic therapy for advanced HCC [13]and those underwent curative treatment for early-stage HCC [14]. The present study found that low basal IGF-1 levels were associated with advanced HCC, such as multiple tumors and advanced stage, and low IGF-1 levels predicted shorter TTP and OS in patients treated with TACE. Collectively, these findings suggest that the association between low circulating IGF-1 and unfavorable outcome may remain consistent across various stages of HCC and treatment modalities, and the oncogenic effects of circulating IGF-1 may not play a determinant role in the progression of HCC. However, the possibility that the autocrine/paracrine effects of IGF-1 may be more important than the systemic effects on HCC cannot be excluded.

The decrease of circulating IGF-1 in patients with cirrhosis or HCC has been attributed to a result of liver damage because hepatocytes are the main contributors of IGF-1 [12]. The findings that serum IGF-1 levels in patients with chronic hepatitis C negatively correlated with histologic fibrosis score and improved during anti-viral therapy may support this hypothesis [10]. Moreover, recent studies showed that IGF-1 replacement or gene transfer therapy induced cytoprotective and anti-inflammatory effects leading to improvement of hepatic fibrosis in cirrhotic rats [26], [27], and IGF-1 treatment improved serum albumin levels in patients with cirrhosis [28]. These findingsimply that low IGF-1 level is not only a result of liver damage, but may be also a contributor to the development of cirrhotic features by promoting pro-inflammatory and profibrogenicresponses. Because inflammatory microenvironmentand advanced hepatic fibrosis/cirrhosis are important in hepatocarcinogenesis [29]–[31], these maycontribute to the higher recurrence rates in patients with low IGF-1 levels, althoughour hypothesis could not be directly tested in the present study because histological data were unavailable. Despite most of the patients in our study had preserved Child-Pugh class A liver function, the levels of IGF-1 were still useful predictors of progression and death, independent of remnant liver function. Therefore, the levels of serum IGF-1, expressed as continuous value, may be helpful for accurately assessing hepatic function and the prognostic stratification of patients with HCC in combination with traditional stepwise parameters, such as Child-Pugh class or BCLC stage.

The present study has some limitations. First, the study was performed at a single institution, and the cut-off level of IGF-1 (125 ng/mL) that was used to divide the patient population was different from previous cohort studies [13], [25]. The difference may be attributable to several factors, including different ethnicity (i.e., Asians), a relatively low proportion of patients with chronic hepatitis C in our cohort, and different purposes and methods for cut-off determination. However, when analyzed as a continuous value, IGF-1 level was significantly associated with disease progression and patient survival. Thus, its prognostic value appears to be consistent, regardless of the difference in the cut-off values. Further studies will be needed to develop multi-stage stratification that can discriminate subgroups with different prognoses. Second, the proportion of patients with very-early- or early-stage HCC was relatively high. Therefore, our study may lack a representative sampling of patients with intermediate-to-advanced HCC, the standard target of TACE. However, the focus of our study was to evaluate the prognostic value of IGF-1 in overall HCC patients who were initially treated by TACE. Therefore, we did not exclude patients with very-early- or early-stage HCC who were unsuitable for curative therapies and thereby underwent TACE. Third, the influence of IGF-1 on OS was not fully investigated because of the relatively short follow-up period and small number of events. Further follow-up of our cohort will clarify this association.

In conclusion, the present prospective study found a significant association between serum IGF-1 levels and treatment outcome in HCC patients who underwent TACE. Thus, serum IGF-1 levels may serve as an indicator of liver function and prognostic marker that reflects TTP and OS in HCC patients, which will be helpful for the precise risk stratification of patients.
